# Acaricidal efficacy of a new combination of fipronil and permethrin against *Ixodes ricinus* and *Rhipicephalus sanguineus* ticks

**DOI:** 10.1186/s13071-015-0681-0

**Published:** 2015-01-27

**Authors:** Pascal Dumont, Theodore S Chester, Boyd Gale, Mark Soll, Josephus J Fourie, Frédéric Beugnet

**Affiliations:** Merial S.A.S., 29 Av. Tony Garnier, 69007 Lyon, France; Merial Limited, 3239 Satellite Blvd., Duluth, GA 30096 USA; Charles River Laboratories Preclinical Services Ireland Ltd., Carrentrila, Ballina, Co. Mayo, Ireland; ClinVet International (Pty) Ltd, PO Box 11186, 9321 Universitas, Bloemfontein, South Africa

**Keywords:** Ticks, *Ixodes ricinus*, *Ripicephalus sanguineus*, Permethrin, Fipronil, Dog, Acaricide, Frontline Tri-Act®/Frontect®

## Abstract

**Background:**

Two blinded, controlled laboratory studies were conducted to assess the acaricidal efficacy of a new combination of fipronil and permethrin (Frontline Tri-Act®/Frontect®) against two tick species. Study A evaluated the efficacy of the product against both *Ixodes ricinus* and *Rhipicephalus sanguineus* and Study B evaluated the efficacy against *R. sanguineus* only.

**Methods:**

16 (Study A) and 12 (Study B) healthy adult dogs were allocated to two groups in each study. Dogs in Group 1 served as untreated controls. Dogs in Group 2 were treated with a new topical spot-on formulation containing 6.76% (w/v) fipronil + 50.48% (w/v) permethrin once on Day 0. Each dog of study A was infested with 50 unfed adult ticks of each species and each dog of study B was infested with 50 unfed adult *Rhipicephalus sanguineus* prior to treatment (Day −2 in Study A, Day −1 in Study B) and post treatment on Days 7, 14, 21 and 28. The ticks were removed and counted 48 h after treatment (Day 2) or subsequent infestations (Days 9, 16, 23 and 30). Acaricidal efficacy was defined as the percent reduction in the number of live ticks in the treated group compared to the untreated control group.

**Results:**

The percent efficacy in the treated group for *R. sanguineus* was 100%, 100%, 100%, 100% and 96.7% in Study A, and 94.4%, 100%, 100%, 98.7% and 98.0% in Study B, for counts performed on Days 2, 9, 16, 23 and 30, respectively. For *I. ricinus*, in Study A, the percent efficacy of the treatment was 100%, 100%, 100%, 100% and 99.2% for counts performed on Days 2, 9, 16, 23 and 30, respectively. There was a significant difference of the geometric mean numbers of live ticks between the treated and control groups at each time point in each study (*p* = 0.005 for every day in Study A, and *p* < 0.005 for every day in Study B).

**Conclusions:**

A single topical administration of a combination of fipronil and permethrin provides excellent acaricidal efficacy against both *I. ricinus* and *R. sanguineus* for at least 4 weeks.

## Abrégé

**Contexte:**

Deux études expérimentales contrôlées et randomisées ont été conduites afin de mesurer l’efficacité acaricide d’une nouvelle combinaison de fipronil et de perméthrine (Frontline Tri-Act®/Frontect®) contre deux espèces de tiques. L’étude A a évalué l’efficacité du produit vis-à-vis de *Ixodes ricinus* et *Rhipicephalus sanguineus* et l’étude B a évalué l’efficacité vis-à-vis de *R. sanguineus* seul.

**Méthodes:**

Pour chaque étude, 16 (étude A) et 12 (étude B) chiens adultes en bonne santé ont été répartis en deux groupes. Les chiens du groupe 1 servaient de témoins non traités. Les chiens du groupe 2 étaient traités une fois au jour 0 avec une nouvelle formulation spot-on topique contenant 6,76% (w/v) de fipronil + 50,48% (w/v) de perméthrine. Chaque chien de l’étude A a été infesté par 50 tiques adultes à jeun de chaque espèce et chaque chien de l’étude B a été infesté par 50 *Rhipicephalus sanguineus* adultes à jeun, avant le traitement (jour −2 dans l’étude A, jour −1 dans l’étude B) et après traitement, aux jours 7, 14, 21 et 28. Les tiques ont été retirées et comptées 48 h après traitement (jour 2) ou après chaque infestation (jours 9, 16, 23 et 30). L’efficacité acaricide est définie comme le pourcentage de réduction du nombre de tiques vivantes dans le groupe traité par rapport au groupe témoin non traité.

**Résultats:**

Le pourcentage d’efficacité contre *R. sanguineus* dans le groupe traité a été de 100%, 100%, 100%, 100% et 96,7% dans l’étude A, et 94,4%, 100%, 100%, 98,7% et 98,0% dans l’étude B, pour les comptages réalisés aux jours 2, 9, 16, 23 et 30, respectivement. Pour *I. ricinus*, dans l’étude A, le pourcentage d’efficacité du traitement était de 100%, 100%, 100%, 100% et 99,2% pour les comptages réalisés aux jours 2, 9, 16, 23 et 30, respectivement. Une différence significative entre les moyennes géométriques de tiques vivantes entre les groupes traité et témoin, a été retrouvée à chaque point de comptage dans les deux études (p = 0,005 pour chaque jour dans l’étude A, et p < 0,005 pour chaque jour dans l’étude B).

**Conclusions:**

Une administration topique unique de la combinaison fipronil et perméthrine offre une excellente efficacité acaricide à la fois contre *I. ricinus* et *R. sanguineus* pour au moins 4 semaines.

## Background

Tick infestations represent an important problem for dogs and their owners. *Ixodes ricinus* and *Rhipicephalus sanguineus* are two of the most common tick species infesting dogs in Europe. They are known to also infest humans, and are vectors for several important canine and human diseases’ agents [1,2]. Adequate control measures to prevent and treat infestations in dogs against these species are therefore very important for the health and well-being of dogs.

*Ixodes ricinus* is widely distributed throughout Europe [3] and is the most common tick species in northwestern Europe [4]. The distribution of this tick species has been expanding in both range and period of activity, possibly due to climate change [5-7]. In dogs, *I. ricinus* is the vector of *Anaplasma phagocytophilum, Borrelia burgdorferi* sensu lato and tick borne encephalitis (TBE) virus [3]. In humans, it can serve as the vector for *B. burgdorferi* s.l., *Babesia divergens*, *Babesia microti* and also TBE virus [1]. *Rhipicephalus sanguineus* has a worldwide distribution and is the most commonly encountered tick species (or complex of species) infesting dogs [8]. It is the vector for the most common tick-borne pathogens in dogs, which include *Ehrlichia* spp.*, Rickettsia* spp.*, Babesia* spp. and *Hepatozoon canis.* [3,8-10], as well as for the pathogens of spotted fever and ehrlichiosis in humans.

Adequate control of tick infestations on dogs is important for both the health of the dog and for preventing pet dogs from carrying and serving as a source of ticks in the home environment of their owners. Various formulations of topical- and collar-based treatments have been employed as a strategy to combat tick infestations in dogs [8,11-13]. Fipronil is one of the most widely used insecticides/acaricides to control fleas and ticks on both dogs and cats [13]. Pyrethroids have also been used as insecticides and acaricides with repellent activity on dogs [14-18], production animals [19] and humans [20,21]. Frontline Tri-Act®/Frontect® is a novel combination of 6.76% w/v fipronil and 50.48% w/v permethrin that has been developed as a monthly topical solution for dogs to provide broad spectrum ectoparasite control. Studies were conducted to confirm the acaricidal efficacy of this combination against *I. ricinus* and *R. sanguineus* ticks.

## Methods

The studies were designed in accordance with the “World Association for the Advancement of Veterinary Parasitology (W.A.A.V.P.) guidelines for evaluating the efficacy of parasiticides for the treatment, prevention and control of flea and tick infestation on dogs and cats” [22] and were conducted in accordance with Good Clinical Practices (GCP) as described in the International Cooperation on Harmonisation of Technical Requirements for Registration of Veterinary Medicinal Products (VICH guideline 9).

### Animals

None of the dogs had been exposed to ectoparasiticides for the three months preceding the study. Prior to allocation for each study, 20 healthy Beagle dogs (in Study A) and 14 mixed breed dogs (in Study B) were infested with approximately 50 +/−5 unfed adult *R. sanguineus* (approx. equal sex ratio) and the ticks were removed and counted from each dog after 48 h. The two male and two female dogs with the lowest tick counts were dropped from Study A and the two dogs (regardless of sex) with the lowest tick counts were dropped from Study B. The remaining dogs were ranked within sex by descending tick counts and assigned to blocks of two dogs each. Within blocks, each dog was randomly allocated to the treated and untreated groups. The dogs were managed with due regard for their well-being in accordance with Merial, South African and Irish Institutional Animal Care and Use Committee requirements. The dogs were housed individually. A veterinary examination performed prior to the start of each study ensured that all dogs were healthy and suitable for inclusion, and the dogs were observed daily for any health changes throughout the study.

### Treatment

Dogs in Group 1 of Study A and B served as untreated controls. Dogs in Group 2 of both studies were treated once on Day 0 with a topical formulation containing 6.76% (w/v) fipronil and 50.48% (w/v) permethrin with a total volume corresponding to the appropriately sized pipette based upon body weight such that dogs weighing less than or equal to 10 kg received 1.0 mL, dogs weighing greater than 10 but less than 20 kg received 2.0 mL, and dogs weighing greater than 20 but less than 40 kg received 4.0 mL. The total volume of the product was divided into two approximately equal fractions and placed on the skin on the midline of the neck. One fraction was applied between the base of the skull and the shoulder blades and the other was applied at the front of the shoulder blades. All of the animals were observed hourly for any adverse reaction for 4 h following the treatment of the last animal.

### Ticks

The ticks used in the studies were unfed adult *I. ricinus* ticks (50 females with 4–5 added males for each challenge) and unfed adult *R. sanguineus* ticks (approx. equal sex ratio) that were not known to be resistant to any ectoparasiticide. The ticks originated from European tick populations, now bred under experimental conditions. The *I. ricinus* ticks originated from natural populations from the United Kingdom, Slovakia, and Ireland. The *R. sanguineus* ticks used in Study A originated from natural populations from Oxford, UK, and for Study B the ticks originated from field collections in France.

### Tick infestation and counting

Dogs in both studies were infested prior to treatment (Day −2 in Study A, Day −1 in Study B) and post treatment on Days 7, 14, 21 and 28 with 50 unfed adult *R. sanguineus* (approx. equal sex ratio). In Study A each dog was also infested with 50 unfed adult female *I. ricinus* ticks (with at least an additional 10 male *I. ricinus* ticks to stimulate female attachment) at the same time points.

Live ticks on the dogs were removed and counted on Days 2, 9, 16, 23 and 30 (48 h after treatment or infestation). *Ixodes ricinus* and *R. sanguineus* ticks were counted and recorded separately. Only female *I. ricinus* were counted while for *R. sanguineus,* both females and males were counted.

For tick infestations and counting in Study A, dogs were anesthetized with intramuscular injections of ketamine (Narketan®, Vetoquinol; approx. 10.0 mg/kg) and xylazine (Chanazine®, Chanelle; approx. 2.0 mg/kg). In Study B, dogs were sedated with medetomidine (Domitor®, Pfizer; 0.06 mg/kg) for tick infestations only.

### Data analysis

For each tick species, total counts of live ticks were transformed to the natural logarithm of (counts + 1) for calculation of geometric means (GM) by treatment group at each time point. As described in the WAAVP guidelines, the use of geometric means allow to describe a central tendancy whereas arithmetic means maintains the same weight to extreme data. Percent efficacy of the treated group compared to the control group was calculated at every post-treatment time point using the formula 100×[(C-T)/C], where C is the GM for the control group and T is the GM for the treated group.

In Study A the treated group was compared to the control group at every post-treatment time using the Friedman rank test with blocks defined as the allocation blocks. The testing was two-sided and used a significance level of 5%. All analyses were performed using SAS® Version 9.1.3. In Study B the groups were compared by a non-parametric analysis using the Mann–Whitney test. SAS® Version 9.3 TS Level 1 M2 was used for the statistical analysis.

## Results

No adverse reactions to treatment were observed in any dog in either study, including during the 4 h after treatment.

A summary of the tick counts and efficacy results are shown in Table 1 (Study A) and Table 2 (Study B).

**Table 1:** **Efficacy of a new combination of fipronil and permethrin against** ***Rhipicephalus sanguineus*** **and** ***Ixodes ricinus*** **(Study A)**

**Tick species**	**Study day**	**Geometric mean of live ticks**		
		**Untreated control dogs (n = 8)**	**Treated dogs (n = 8)**	**Efficacy (%)**
*R. sanguineus*	2	31.8	0.0	100.0^*^
*R. sanguineus*	9	24.8	0.0	100.0^*^
*R. sanguineus*	16	27.8	0.0	100.0^*^
*R. sanguineus*	23	22.8	0.0	100.0^*^
*R. sanguineus*	30	24.7	0.7	96.7^*^
*I. ricinus*	2	36.7	0.0	100^*^
*I. ricinus*	9	32.5	0.0	100.0^*^
*I. ricinus*	16	30.7	0.0	100.0^*^
*I. ricinus*	23	31.6	0.0	100.0^*^
*I. ricinus*	30	33.3	0.3	99.2^*^

*Significant difference between the tick population means of the treated and control groups (*p* = 0.005).

**Table 2:** **Efficacy of a new combination of fipronil and permethrin against** ***Rhipicephalus sanguineus*** **(Study B)**

**Study day**	**Geometric mean of live ticks**		
	**Untreated control dogs (n = 6)**	**Treated dogs (n = 6)**	**Efficacy (%)**
2	35.9	2.0	94.4^*^
9	27.0	0.0	100.0^*^
16	33.1	0.0	100.0^*^
23	38.7	0.5	98.7^*^
30	32.0	0.6	98.0^*^

*Significant difference between the tick population means of the treated and control groups (*p* ≤ 0.005).

The percent efficacy of the treated group for *R. sanguineus* was 100%, 100%, 100%, 100% and 96.7% in Study A, and 94.4%, 100%, 100%, 98.7% and 98.0% in Study B, for counts performed on Days 2, 9, 16, 23 and 30, respectively. There was a significant difference of the geometric mean number of live ticks between the treated and control groups in both studies at each time point (*p* = 0.005 for every day in Study A, and *p* < 0.005 for every day in Study B).

For *I. ricinus* the percent efficacy of the treated group was 100%, 100%, 100%, 100% and 99.2% for counts performed on Days 2, 9, 16, 23 and 30, respectively. There was a significant difference of geometric mean number of live ticks between the treated and control groups at each time point (*p* = 0.005 for every day).

In addition, the majority of dogs remained free of live ticks in the treated groups. All of the dogs treated with the tested spot-on were not infested with ticks at the counts performed for *I. ricinus* on Days 2, 9, 16 and 23; on Day 30, 6 out of 8 treated dogs did not harbour tick (data not shown). In Study A, no ticks were found on all of the treated dogs at the counts performed for *R. sanguineus* on Days 2, 9, 16 and 23; on Day 30, only 2 of 8 dogs were found to be infested with ticks. In Study B all of the dogs in the treated group were not infested with ticks on Days 9 and 16. On Days 23 and 30, 4 out of 6 treated dogs were tick free (data not shown).

## Discussion

The results of the studies presented here demonstrate that a single topical treatment with the combination of fipronil and permethrin provides excellent efficacy against both *I. ricinus* and *R. sanguineus.* In each counting time points in both studies, the mean infestation rate in the control group for both *I. ricinus* and *R. sanguineus* was very good. The average number of *I. ricinus* per dog in the control group ranged from 30.7 to 36.7 ticks per dog, while for *R. sanguineus* the average tick number per dog ranged from 22.8 to 31.8 in Study A and from 27.0 to 38.7 in Study B. In comparison, there were no live ticks found on the dogs treated with the combination of fipronil and permethrin in either study for the Day 9 and Day 16 counts and only a very low number of ticks found on Days 2, 23, and 30 (Tables 1 and 2). These results are in the range to what has already been published with other acaricidal spot-on formulations like Frontline® Combo or Advantix® [8,12,13,16-18] and exceed the European regulatory threshold of more than 90% of efficacy counted at 48 h to get a claim.

Frontline Tri-Act®/Frontect® has also been shown to have repellent and parasiticidal efficacy against *Dermacentor* ticks [23], fleas [24,25], mosquitoes and plebotomine sandflies [26,27], indicating that the combination of fipronil and permethrin can be an important component in the reduction of the risk of transmission of most canine vector-borne diseases.

## Conclusions

In conclusion, a single topical administration of a combination of fipronil and permethrin provides excellent acaricidal efficacy against both *I. ricinus* and *R. sanguineus* for at least 4 weeks. The product is safe and can also be used to reduce the risk of transmission of tick-borne pathogens in dogs.
